# A comparative retrospective analysis: myocutaneous flap versus skin flap in V-Y medial epicanthal fold reconstruction

**DOI:** 10.3389/fsurg.2024.1335796

**Published:** 2024-02-29

**Authors:** Bulin Wang, Shengchang Zhang, Yi Chen, Zhihong Liu, Jiangang Yu, Huimin Zhou, Er Pan

**Affiliations:** ^1^ Department of Aesthetic Plastic Surgery, Guangzhou Eye-Nose-Fat Aesthetic Plastic Surgery Hospital, Guangzhou, China; ^2^ Aesthetic Medical School, Yichun University, Yichun, China; ^3^ Department of Obstetrics and Gynecology, Guangzhou Women and Children’s Medical Center, Guangzhou, China

**Keywords:** myocutaneous flap V-Y procedure, flap-only V-Y procedure, medial epicanthal fold reconstruction, epicanthoplasty, comparative retrospective analysis

## Abstract

**Objectives:**

To evaluate the comparation of myocutaneous flap vs. skin flap in V-Y medial epicanthal fold reconstruction.

**Methods:**

The study, conducted from April 2017 to June 2022, involved two groups: group A, comprising 21 patients who underwent medial epicanthal fold restoration surgery using the V-Y advancement method with a skin flap, and group B, comprising 83 patients who underwent the same procedure, while with a myocutaneous flap for orbicularis oculi ring reconstruction. Intercanthal distances were measured preoperatively, recorded during preoperative and postoperative reviews, and assessed through a 4-point Likert satisfaction questionnaire.

**Results:**

A total of 104 patients were followed up for 6 months postoperatively. In group A, preoperative intercanthal distances ranged from 28.7 mm to 38.2 mm, increasing to 30.2 mm–40.6 mm postoperatively, with a mean increase of 3.0 mm (*P* < 0.05). In group B, preoperative distances ranged from 28.8 mm to 38.0 mm, increasing to 32.2 mm–41.5 mm postoperatively, with a mean increase of 3.9 mm (*P* < 0.05). Group B exhibited a higher overall satisfaction rate compared to group A.

**Conclusion:**

The myocutaneous flap V-Y procedure, employing the principle of orbicularis oculi ring reconstruction, achieves more stable postoperative results than the flap-only V-Y procedure. Consequently, it can be regarded as the preferred surgical technique.

## 1 Introduction

The presence of a medial epicanthal fold, also known as Mongoloid fold or Mongolian wrinkles, is attributed to the overdevelopment of the orbicularis oculi and related structures (1), predominantly observed in Asians. Recent shifts in aesthetic preferences have led to concerns that a medial epicanthal fold might give the eyes a smaller or less refined appearance. Consequently, many individuals choose epicanthoplasty to correct this feature. Several studies on epicanthoplasty have been conducted, indicating successful outcomes (2–10). However, practical experience has shown complications such as excessive exposure of the lacrimal caruncle, conjunctival ectropion, and scar contracture due to overcorrection, increasing the demand for medial epicanthal fold reconstruction. To date, prior techniques for medial epicanthal fold restoration, specifically the reverse skin redraping method and the V-Y method/modified V-Y method, have been inadequately documented (11–17). While these studies detailed the skin flap design, they often overlooked the significance of deep skin orbicularis oculi reconstruction. This oversight could lead to complications, such as incision dehiscence, under-correction, and scar formation. In this retrospective study, a comparison was made between the myocutaneous flap V-Y procedure and the skin flap-only V-Y procedure.

## 2 Material and methods

### 2.1 Patients’ selection and group comparability

Between April 2017 and June 2022, patients, with a mean age of 28 years (range: 19–56 years), underwent medial epicanthal restoration surgery at Guangzhou Yanmeihui Medical Aesthetic Clinic. All participants had previously undergone epicanthoplasty, resulting in an excessively wide intercanthal distance, and had their initial surgery more than 6 months before their participation in this study. Preoperative measurements, including intercanthal distance, were recorded. The study protocol adhered to the Declaration of Helsinki guidelines, and both the doctor and each patient signed an informed consent form before the surgery. Patients eligible for this study had previously undergone epicanthoplasty, resulting in an excessively wide intercanthal distance. All patients included in the study had completed the initial surgery more than 6 months before recruitment, ensuring a stable postoperative period. To assess comparability between group A and group B, preoperative patient status, including complications from prior surgeries, was thoroughly evaluated. Patients with complications that could potentially impact the study outcomes were excluded. This strict selection criterion ensured a uniform preoperative patient status in both groups.

### 2.2 Ethical approval

All procedures performed in this study involving human participants were in accordance with the ethical standards and the Declaration of Helsinki (1964) and its later amendments. Appropriate ethics approval was obtained from the Institutional Review Board of Yichun University (Yichun, China).

### 2.3 Surgical technique

**Figure 1:**
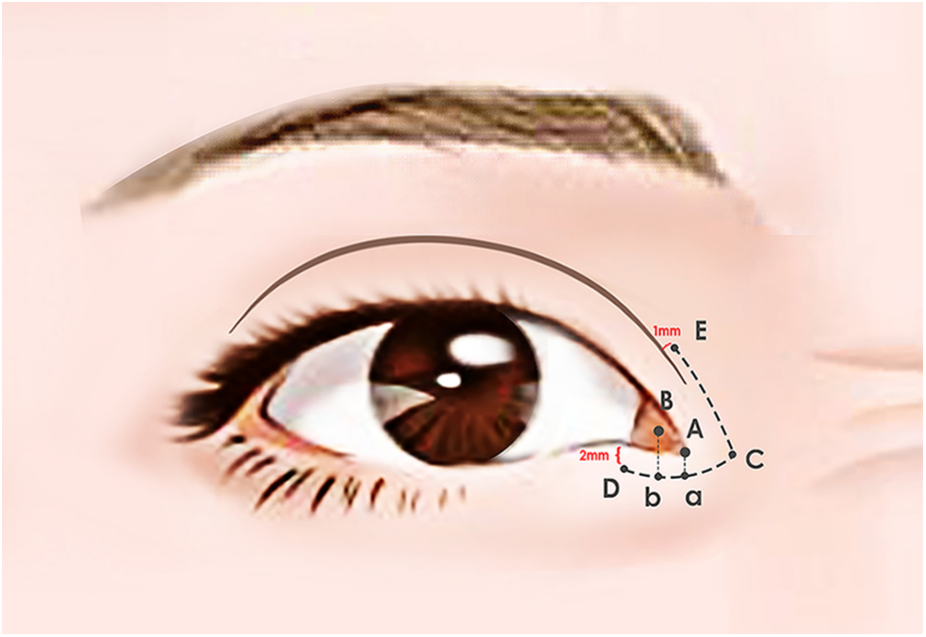
Surgical markings. Point A was marked about 2 mm from the lower lid margin, perpendicular to the medial canthal point (**A**) For a desired 2 mm reduction in the medial canthal distance, point B (b) was positioned laterally to point A(a), maintaining AB = ab = 2 mm, while point b was also situated 2 mm from the lower lid margin. Point C (AB: AC = 1:2) was delineated 4 mm medially from point a along the lower lid margin. Point D was approximately 2 mm lateral to point b. These points, D, b, a, and C, were connected sequentially, forming a smooth arc, equidistant from the lower lid margin, creating the DC arc. Point E was marked on the upper lid, approximately 1 mm above the double eyelid line. Once again, connecting points E and C formed an arc, ensuring CD was equidistant from CE.

Flap design: The patient assumed a supine position. Initially, point A was marked about 2 mm from the lower lid margin, perpendicular to the medial canthal point (A). For a desired 2 mm reduction in the medial canthal distance, point B (b) was positioned laterally to point A(a), maintaining AB = ab = 2 mm, while point b was also situated 2 mm from the lower lid margin. Point C (AB: AC = 1:2) was delineated 4 mm medially from point a along the lower lid margin. Point D was approximately 2 mm lateral to point b. These points, D, b, a, and C, were connected sequentially, forming a smooth arc, equidistant from the lower lid margin, creating the DC arc. Point E was marked on the upper lid, approximately 1 mm above the double eyelid line. Once again, connecting points E and C formed an arc, ensuring CD was equidistant from CE. These line segments constituted the two arms of the V-shaped incision (Figure 1).

**Figure 2:**
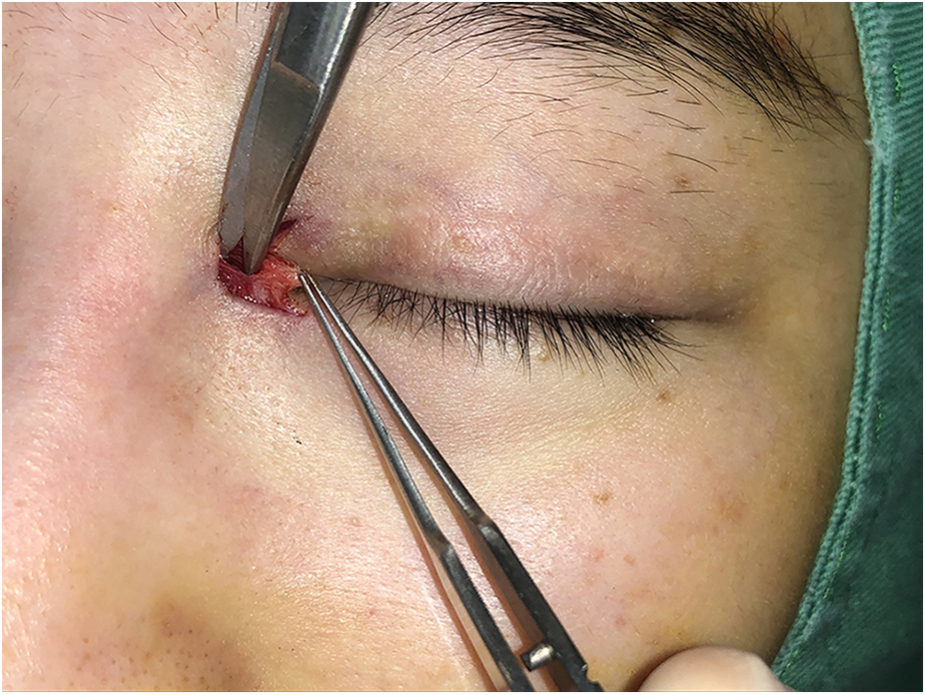
Separation under the orbicularis oculi muscle. In group B, the incision was made and separated beneath the orbicularis oculi muscle.

**Figure 3:**
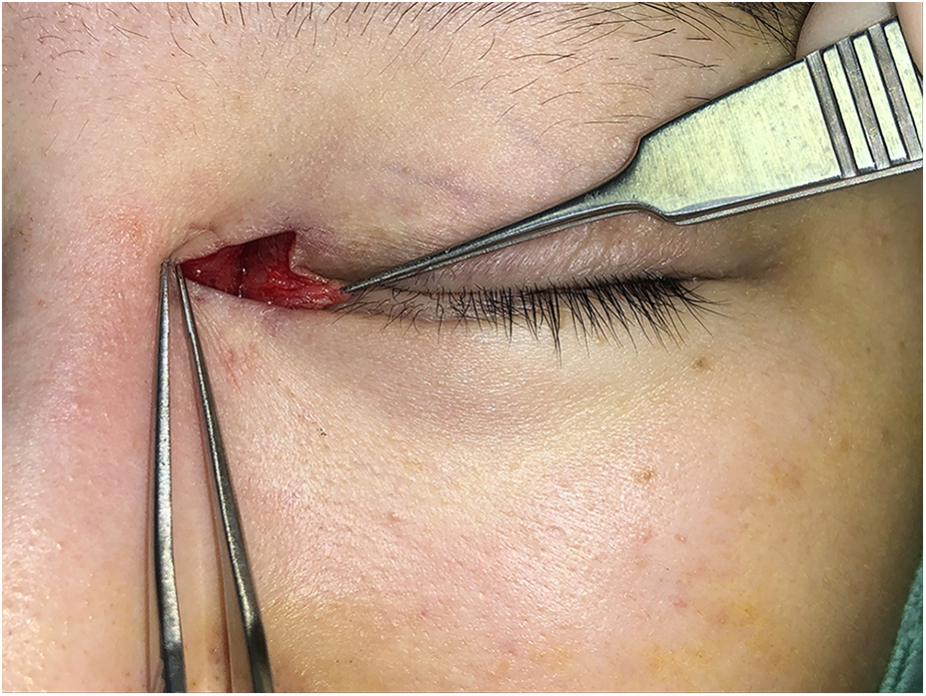
Creation of a peel to lift the orbicularis oculi flap. A full peel was performed to elevate the orbicularis oculi flap.

**Figure 4:**
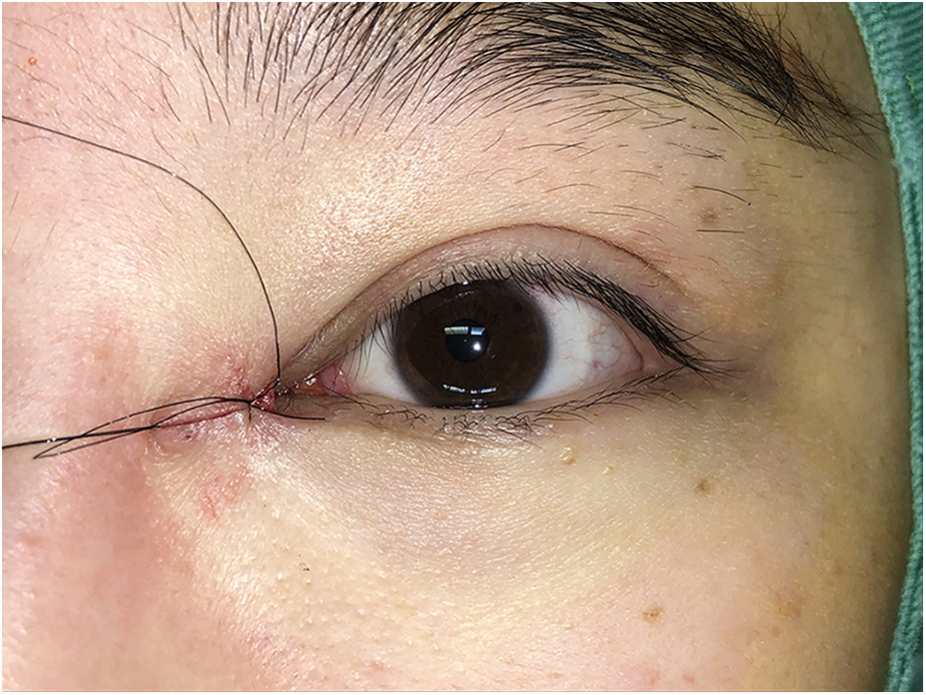
Suturing the full layer of the orbicularis oculi at point b for orbicularis oculi ring reconstruction with a 6-0 nylon thread. The skin of the medial epicanthal fold was pulled towards the nasal side, and suturing began at point b, where the orbicularis oculi was first sutured with a 6-0 nylon thread to the full layer of the corresponding location on the upper lid, marking the starting point for orbicularis oculi ring reconstruction.

**Figure 5:**
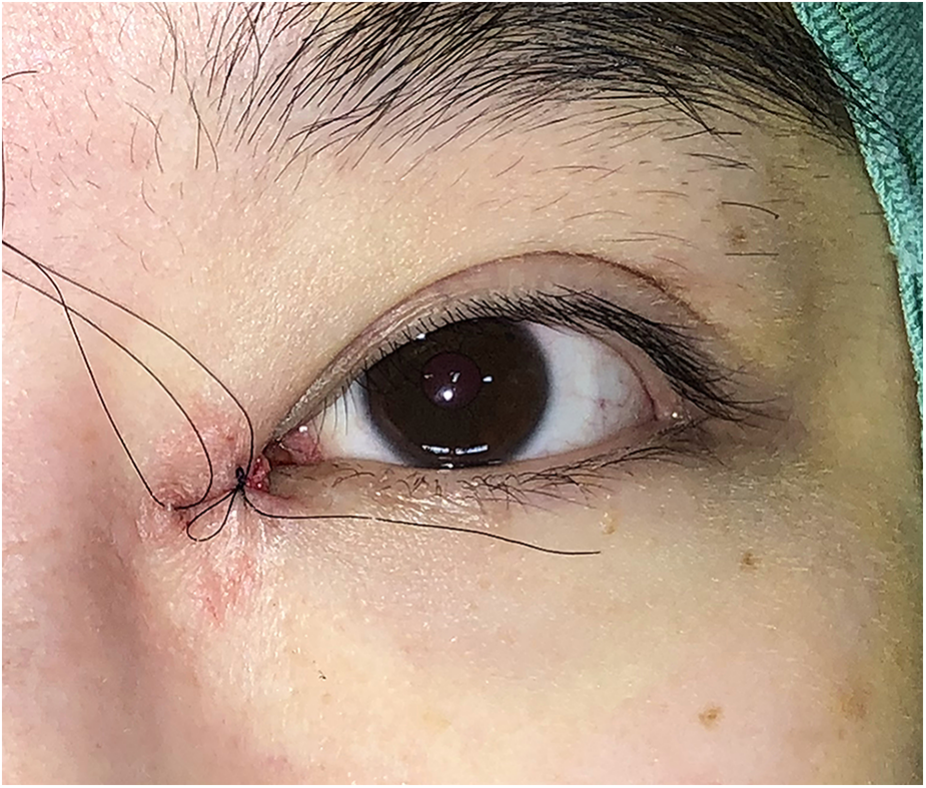
Suturing the orbicularis oculi under the skin of the Y flap with a 6-0 PDS thread to complete the orbicularis oculi ring reconstruction. A 6-0 PDS thread was used for intermittent suturing of the orbicularis oculi beneath the skin of the Y flap.

**Figure 6:**
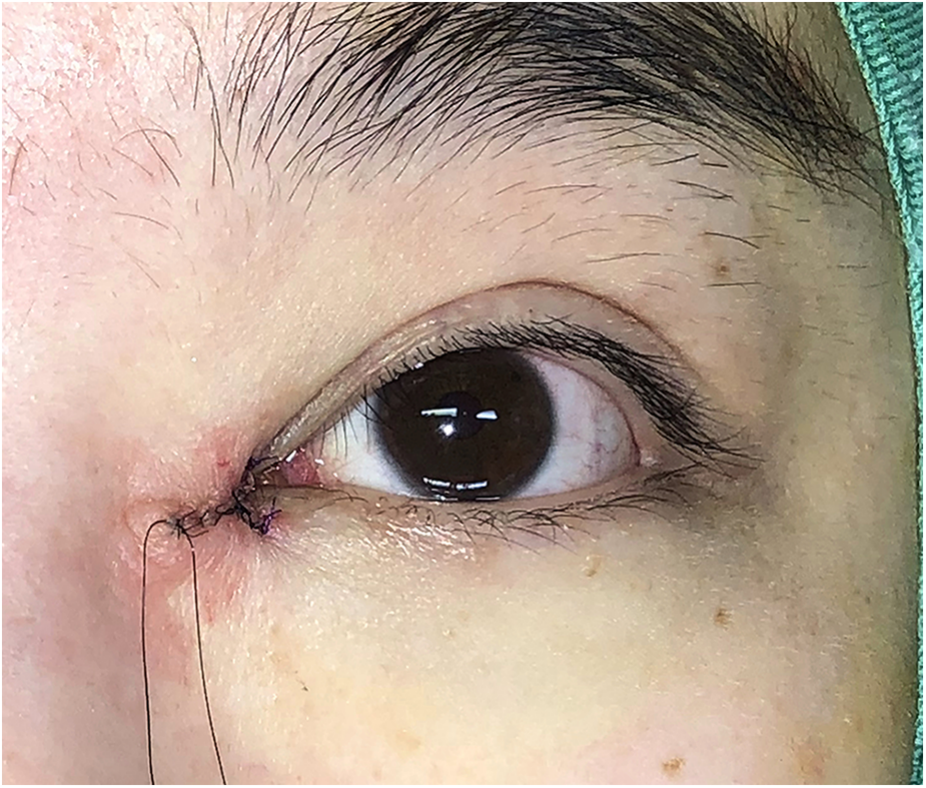
Suturing the skin in a Y-shape to obtain eversion of the incision with an 8-0 nylon thread. The V-shaped flap was flipped to form the anterior surface of the new fold. The skin above and below the incision was sutured interruptedly with an 8-0 nylon thread in a Y-shape to achieve eversion of the incision.

Surgical procedures: All surgeries were conducted under local anesthesia using 2% lidocaine and 1:100,000 epinephrine. The skin was incised along the marked line. In group B, the incision was made and separated beneath the orbicularis oculi muscle (Figure 2). At this level, a full peel was performed to elevate the orbicularis oculi flap (Figure 3), extending up to the orbital orbicularis muscle. The skin of the medial epicanthal fold was pulled towards the nasal side, and suturing began at point b, where the orbicularis oculi was first sutured with a 6-0 nylon thread to the full layer of the corresponding location on the upper lid, marking the starting point for orbicularis oculi ring reconstruction (Figure 4). Subsequently, a 6-0 PDS thread was used for intermittent suturing of the orbicularis oculi beneath the skin of the Y flap (Figure 5). This ensured a close match between the orbicularis oculi above and below the incision, completing the orbicularis oculi ring reconstruction. The V-shaped flap was then flipped to form the anterior surface of the new fold. The skin above and below the incision was sutured interruptedly with an 8-0 nylon thread in a Y-shape to achieve eversion of the incision (Figure 6). The surgical details are shown in Supplementary file 1 (surgical video).

In group A, dissection occurred in the subcutaneous plane, without dissecting the orbicularis oculi. Only the skin flap was turned over and sutured with a nylon thread in a Y-shape. In both groups, the surgical area was bandaged with pressure, and patients were advised to apply local ice for 48 h postoperatively. Incision sutures were removed on the 5th day after surgery. In this study, the ratio of the intercanthal distance to the palpebral width was computed in both groups.

### 2.4 Subjective patient-reported outcomes

All patients underwent a 6-month follow-up, during which the intercanthal distance was re-measured at the postoperative review. Patients’ satisfaction was evaluated using a 4-point Likert scale-based questionnaire. This subjective measure allowed us to capture patients’ individual perceptions and experiences following the medial epicanthal fold restoration surgery. The Likert scale is a well-established tool in medical research, enabling patients to express their satisfaction levels based on their personal feelings and observations (Table 1).

**Table 1:** 4-point Likert satisfaction questionnaire.

Very satisfied	The degree of restoration of the medial epicanthal fold is accurate, the appearance is natural and realistic when viewed from all angles, and the incision scar is not obvious.
Somewhat satisfied	The degree of restoration of the medial epicanthal fold is accurate, the appearance is natural when viewed from a flat angle, the side view is not natural enough, and the incision scar is not obvious.
Somewhat dissatisfied	The medial epicanthal fold is not adequately restored, the appearance is not natural enough in the flat view, or the incision scar is visible.
Very dissatisfied	The medial epicanthal fold is not adequately restored or is excessively restored, the appearance is unnatural, and the incision scar is obvious.

### 2.5 Objective evaluator assessment

To complement the subjective patient-reported outcomes, objective evaluations were conducted by independent evaluators. These evaluators, experienced in facial aesthetics, assessed standardized photographs of patients taken from multiple angles, including frontal, lateral, and oblique views. The evaluators were blinded to the surgical techniques used and were instructed to assess the outcomes based on predefined criteria, including the symmetry of the epicanthal fold, naturalness of eyelid contour, and overall facial harmony.

### 2.6 Comparative analysis

The comparability between group A and group B was further verified through statistical analysis, confirming the homogeneity of preoperative patient characteristics. This approach provided a robust foundation for the comparison of the two surgical methods, ensuring a fair evaluation of their effectiveness in restoring the medial epicanthal fold.

### 2.7 Statistical analysis

Statistical analysis was conducted using SPSS 20.0 software (IBM, Armonk, NY, USA). Measurement data were presented as mean ± standard deviation, and differences between groups were analyzed using two independent sample *t*-tests. A significance level of *P* < 0.05 was considered statistically significant.

## 3 Results

**Figure 7:**
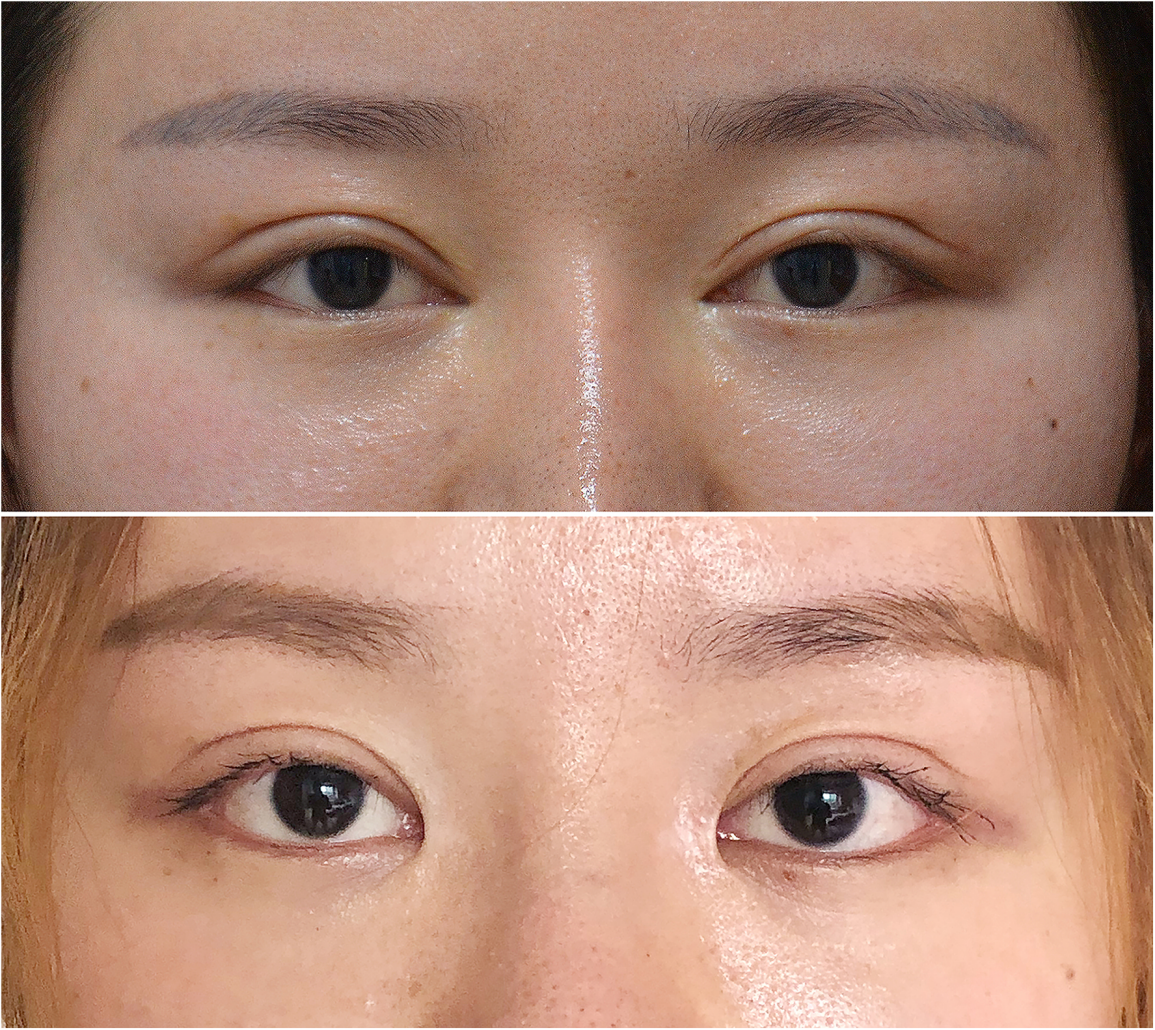
Patient No. 1 in group B. A 27-year-old female patient at 18 months after the surgery (very satisfied). The upper image is presurgical and the lower image is postsurgical.

**Figure 8:**
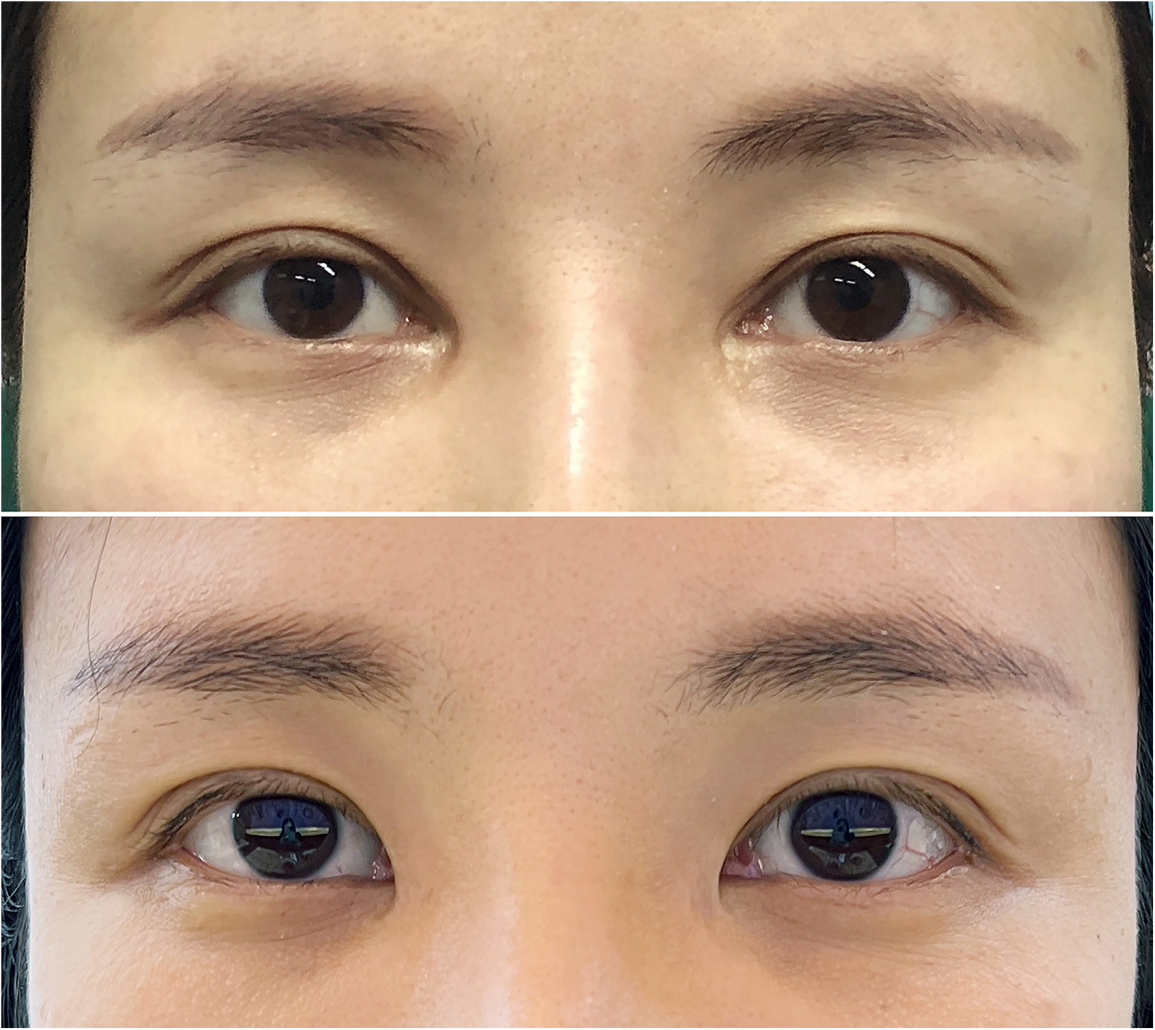
Patient No. 2 in group B. A 36-year-old female patient at 2 years after the surgery (very satisfied). The upper image is presurgical and the lower image is postsurgical.

**Figure 9:**
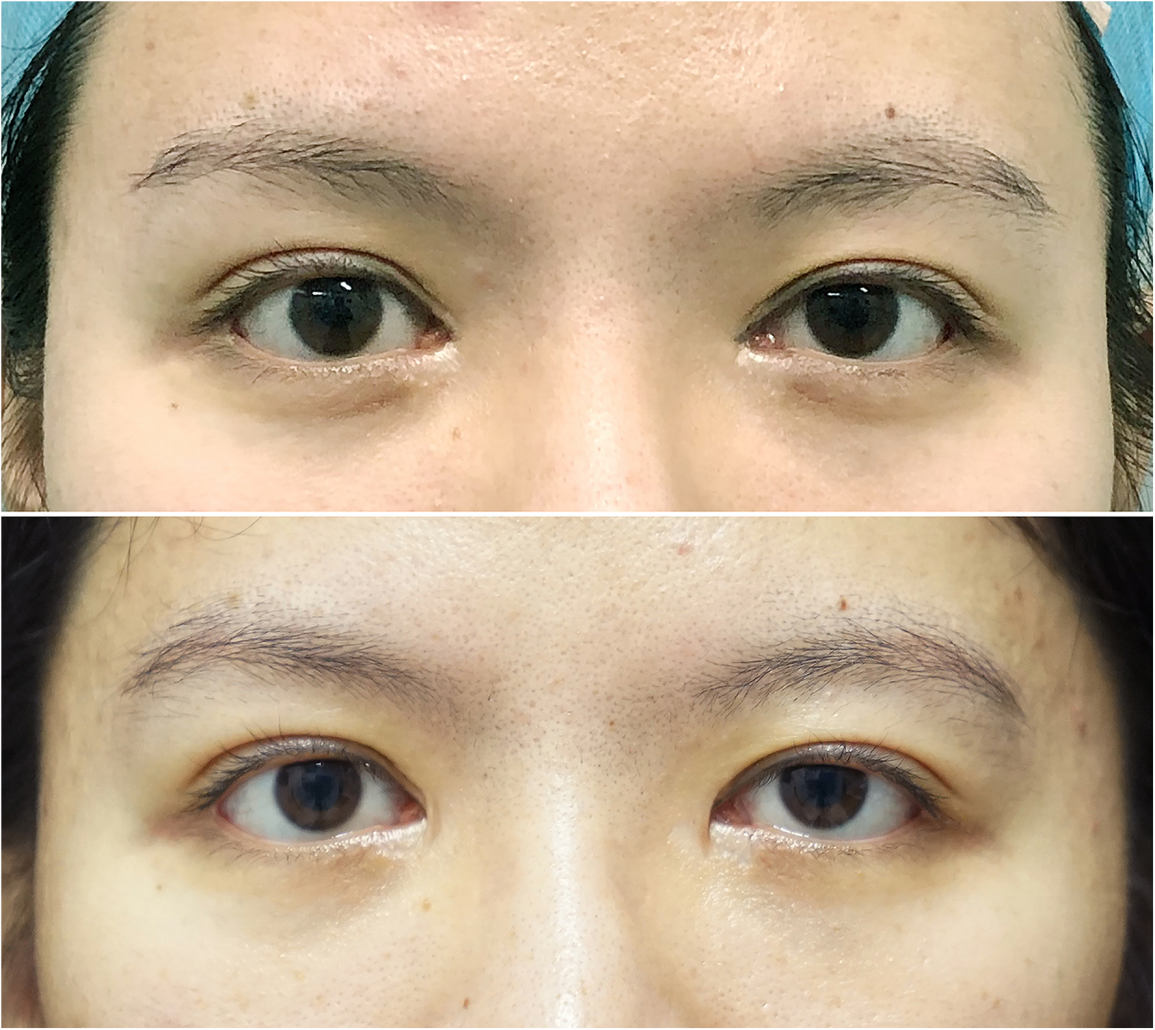
Patient No. 3 in group A. A 27-year-old female patient at 1 year after the surgery, in which the right eye was inadequately corrected (somewhat dissatisfied). The upper image is presurgical and the lower image is postsurgical.

A total of 104 patients (2 males and 102 females, male/female ratio 1/51) underwent medial epicanthal restoration surgery utilizing the V-Y advancement method. In group A, the preoperative intercanthal distance ranged from 28.7 mm to 38.2 mm (mean 33.5 ± 3.4 mm), and postoperatively, it ranged from 30.2 mm to 40.6 mm (mean 36.6 ± 3.2 mm), indicating a mean increase of 3.0 mm (*P* < 0.05). In group B, preoperative intercanthal distance ranged from 28.8 mm to 38.0 mm (mean 33.6 ± 3.3 mm), while postoperative values ranged from 32.2 mm to 41.5 mm (mean 37.5 ± 2.9 mm), reflecting a mean postoperative increase of 3.9 mm compared to the preoperative values (*P* < 0.05). The postoperative intercanthal distance significantly exceeded the preoperative intercanthal distance in both groups, with the increase being greater in group B than that in group A (*P* < 0.05) (Table 2). Moreover, the ratio of the intercanthal distance to the palpebral width was calculated and presented in Table 2. In group A, 14.29% of patients (3/21) reported being very satisfied, 42.86% of patients (9/21) were somewhat satisfied, 19.05% of patients (4/21) expressed somewhat dissatisfaction, and 23.81% of patients (5/21) reported being very dissatisfied. In group B, 34.94% of patients (29/83) were very satisfied, 54.22% of patients (45/83) reported somewhat satisfaction, 6.02% of patients (5/83) expressed somewhat dissatisfaction, and 4.82% of patients (4/83) were very dissatisfied (Table 3). Representative cases are illustrated in Figures 7–9.

**Table 2:** Intercanthal distance and the ratio of intercanthal distance to palpebral width in groups A and B.

	Preoperative measurement, mm	Postoperative measurement, mm	Mean increase in the distance, mm	Preoperative ratio	Postoperative ratio
Group A	28.7–38.2 (mean 33.5 ± 3.4)	30.2–40.6 (mean 36.6 ± 3.2)	3.0 (*P *< 0.05)*	1.16	1.26
Group B	28.8–38.0 (mean 33.6 ± 3.3)	32.2–41.5 (mean 37.5 ± 2.9)	3.9 (*P *< 0.05)*	1.16	1.29

*P < 0.05, compared with preoperative measurement.

**Table 3:** Patient satisfaction.

	Very satisfied	Somewhat satisfied	Somewhat dissatisfied	Very dissatisfied
Group A	14.29% (3/21)	42.86% (9/21)	19.05% (4/21)	23.81% (5/21)
Group B	34.94% (29/83)	54.22% (45/83)	6.02% (5/83)	4.82% (4/83)

During the follow-up evaluation, none of the patients experienced poor incision healing or a hypertrophic scar. There were 8 patients with inadequate correction in group A vs. only 2 patients in group B. In group B, one patient complained of a small intercanthal distance due to overcorrection, whereas no such a complication was found in group A. Besides, 6 patients experienced asymmetry in group A, compared to 3 in group B (Table 4).

**Table 4:** Complications of patients in groups A and B after surgery.

	Inadequate correction	Overcorrection	Asymmetry
Group A	38.10% (8/21)	0% (0/21)	28.57% (6/21)
Group B	2.41% (2/83)	1.20% (1/83)	3.61% (3/83)

### 3.1 Objective evaluations by independent evaluators

To complement the subjective patient-reported outcomes, objective evaluations were conducted by independent evaluators who were experienced in facial aesthetics. They assessed standardized photographs of patients taken from multiple angles, including frontal, lateral, and oblique views. The evaluators were blinded to the surgical techniques used and assessed the outcomes based on predefined criteria.

### 3.2 Symmetry of the epicanthal fold

In both groups A and B, the independent evaluators observed a statistically significant improvement in the symmetry of the epicanthal fold postoperatively. Group A showed an average increase in symmetry of 2.5 points on a scale from 1 to 5 (1 being highly asymmetrical and 5 being perfectly symmetrical). Group B demonstrated an even more pronounced improvement, with an average increase in symmetry of 3.2 points. The difference in improvement between the two groups was not statistically significant.

### 3.3 Naturalness of eyelid contour

The independent evaluators noted a substantial enhancement in the naturalness of eyelid contour in both groups following surgery. Group A exhibited an average increase in naturalness of 2.8 points on a scale from 1 to 5 (1 being highly unnatural and 5 being completely natural). Group B displayed a slightly higher average increase in naturalness, with a gain of 3.0 points. The difference in improvement between the two groups was not statistically significant.

### 3.4 Overall facial harmony

The evaluators found that both groups experienced a noticeable enhancement in overall facial harmony postoperatively. Group A showed an average improvement of 2.7 points on a scale from 1 to 5 (1 being highly disharmonious and 5 being perfectly harmonious). Group B demonstrated a somewhat greater improvement, with an average gain of 3.4 points. The difference in improvement between the two groups was statistically significant, with group B achieving a higher level of overall facial harmony.

## 4 Discussion

In China, epicanthoplasty, frequently performed alongside blepharoplasty, is a popular cosmetic procedure aimed at correcting the medial epicanthal fold. This surgery widens the palpebral fissure, giving the eyes a more elongated appearance and enhancing the smoothness and aesthetics of the eyelid curve. However, the rising prevalence of this surgery has led to an increase in unsatisfactory outcomes. Issues such as excessive exposure of the lacrimal caruncle, conjunctival ectropion, and scar contracture due to overcorrection have caused varying levels of physical and psychological distress to patients, presenting significant challenges to cosmetic surgeons. Regrettably, unlike many epicanthoplasty techniques, restoration surgery for the medial epicanthal fold has been scarcely documented. The V-Y advancement method was first reported by Shin, Y. H. et al. (11), followed by Chung, Y. J. et al., who introduced the reverse skin redraping method and later made improvements to the technique (12, 14).

Our surgical technique is based on the V-Y advancement method. We defined the length of AB as the amount of restoration, used the ECD flap from the lid margin as the new flap, and determined the amount of new flap to be reconstructed by adjusting the distance between these two vertical lines.

The proposed technique simplified the procedure by eliminating the need for a new incision for flap transfer, thereby avoiding additional incisions and tissue dissection. Consequently, it is surgically simpler and potentially reversible. One patient expressed regret after the restoration surgery and opted for reversal the day after the procedure, involving the removal of all sutures and resuturing according to the original incision.

The medial epicanthal fold formation is intricately linked to the preseptal part of the orbicularis oculi muscle that encircles the orbital region and is responsible for eyelid closure (18, 19). Certain epicanthoplasty methods entail dissection or resection of the orbicularis oculi muscle to achieve stable postoperative outcomes. Liu H.P. et al. pointed out that the epicanthal fold results from a mispositioned orbicularis oculi muscle and emphasized the crucial step of releasing skin tension by resecting the orbicularis oculi muscle in correcting the epicanthal fold in Chinese patients. Additionally, Sun W. et al. reported an epicanthoplasty technique based on orbicularis oculi muscle resection. They separated the orbicularis oculi in the medial canthus and excised a triangular flap of muscle tissue to fully alleviate tension (20, 21). Our previous observations revealed that many epicanthoplasty patients exhibit a thin, disrupted, or partially absent medial epicanthal fold, necessitating careful attention during restoration surgery.

In the initial approach, restoration surgery relied solely on a skin flap, neglecting the essential reconstruction of the orbicularis oculi ring. Postoperatively, a tendency toward incision dehiscence was observed, leading to recurrence and resulting in a notably low postoperative satisfaction rate. Between April 2017 and September 2019, 21 procedures were conducted in group A, employing skin flap reconstruction (without a myocutaneous flap) to establish a new medial epicanthal fold. However, the follow-up revealed a satisfaction rate of only 57.15% (comprising very satisfied or somewhat satisfied patients), primarily due to insufficient correction.

This complication was attributed to excessive local tension caused by the absence of a complete orbicularis oculi ring. Consequently, the procedure was modified to incorporate orbicularis oculi ring reconstruction.

In this modified approach, deep plane dissection beneath the orbicularis oculi muscle was crucial, occasionally extending to the orbital region of the orbicularis oculi muscle to enhance local soft tissue mobility. Vertical tissue displacement, specifically, ensured more effective reconstruction of the orbicularis oculi ring. Between October 2019 and June 2022, 83 procedures were performed in group B, utilizing myocutaneous flap reconstruction (not a skin flap) to create a new medial epicanthal fold. Subsequent follow-ups revealed a significantly improved satisfaction rate of 89.16% (consisting of very satisfied or somewhat satisfied patients).

It is important to highlight that most literature reports describe the upper lid incision line as continuous with the double eyelid line. However, based on our clinical observations, we have determined that the line should be positioned approximately 1 mm above the level of the eyelid line. This design aims to create an infold type double eyelid without altering the curvature of the double eyelid line, a feature perceived as a natural double eyelid by the majority of Asians.

Park J.W. et al., in their cadaveric anatomical studies, discovered that individuals with a medial epicanthal fold exhibit a connection between the orbicularis orbitalis septum of the upper and lower eyelids at the medial canthus, a feature absent in individuals without a fold. They proposed that during embryonic development, the eyes move closer, leading to the narrowing and densification of connected orbicularis fibers at the medial canthus, forming the core structure of the medial epicanthal fold19. im, J., et al. emphasized that during V-Y advancement, relying solely on a skin flap may create a cavity (dead space) in the medial canthus. This situation can result in postoperative complications, including poor wound healing, infection, scar contracture, local stiffness, and pain. To address this issue, they utilized an orbicularis muscle strip obtained from the blepharoplasty incision and filled it with an autologous fat tissue graft. Postoperative follow-up results indicated that this method effectively reduced complications and enhanced the restorative outcome (15).

Integrating the findings from the aforementioned studies and our own clinical expertise underscores the critical importance of reconstructing the orbicularis oculi ring in medial epicanthal fold restoration. However, our study has several limitations. Firstly, there is a possibility of recurrence in patients with thin skin and orbicularis muscle if the suture is not adequately tightened. Secondly, individuals with extremely thick skin and orbicularis oculi may experience a newly formed fold that appears thick and differs from the congenital fold, leading to dissatisfaction among some patients. Moreover, due to the varying strength of the orbicularis oculi muscle ring, the new epicanthal fold may exhibit a round shape. Addressing these issues necessitates further exploration and improvement in subsequent studies.

## 5 Conclusions

In conclusion, the V-Y myocutaneous flap advancement method, grounded in the principle of orbicularis oculi ring reconstruction, proves to be significantly more effective than using the skin flap alone for restoring the medial epicanthal fold. Its straightforward design and execution lead to high patient satisfaction rates. This procedure effectively addresses postoperative issues like excessive exposure of the lacrimal caruncle or an overly wide intercanthal distance resulting from overcorrection. Moreover, the incidence of complications such as incisional dehiscence and recurrence is markedly reduced compared to the V-Y advancement method using the skin flap alone. Consequently, this technique stands out as the preferred surgical approach in such cases.

## Data Availability

The raw data supporting the conclusions of this article will be made available by the authors, without undue reservation.
